# A Review of Approaches to the Metallic and Non-Metallic Synthesis of Benzimidazole (BnZ) and Their Derivatives for Biological Efficacy

**DOI:** 10.3390/molecules28145490

**Published:** 2023-07-18

**Authors:** Muhammad Patel, Gopal Avashthi, Amel Gacem, Mohammed S. Alqahtani, Hyun-Kyung Park, Byong-Hun Jeon

**Affiliations:** 1 School of Sciences, P P Savani University, NH 8, GETCO, Near Biltech, Dhamdod, Kosamba, Surat 394125, Gujarat, India; 2 Department of Physics, Faculty of Sciences, University 20 Août 1955 Skikda, Skikda 21000, Algeria; gacem_amel@yahoo.fr; 3 Radiological Sciences Department, College of Applied Medical Sciences, King Khalid University, Abha 61421, Saudi Arabia; mosalqhtani@kku.edu.sa; 4 Bioimaging Unit, Space Research Centre, Michael Atiyah Building, University of Leicester, Leicester LE1 7RH, UK; 5 Department of Pediatrics, Hanyang University College of Medicine, 222 Wangsimni-ro, Seongdong-gu, Seoul 04763, Republic of Korea; neopark@hanyang.ac.kr; 6 Department of Earth Resources and Environmental Engineering, Hanyang University, 222 Wangsimni-ro, Seongdong-gu, Seoul 04763, Republic of Korea

**Keywords:** benzimidazole, heterocyclic derivatives, biological activity

## Abstract

Heterocyclic compounds are significant lead drug candidates based on their various structure–activity relationships (SAR), and their use in pharmaceutics is constantly developing. Benzimidazole (BnZ) is synthesized by a condensation reaction between benzene and imidazole. The BnZ structure consists of two nitrogen atoms embedded in a five-membered imide ring which is fused with a benzene ring. This review examines the conventional and green synthesis of metallic and non-metallic BnZ and their derivatives, which have several potential SARs, along with a wide range of pharmacological properties, including anti-cancer, anti-inflammatory, anti-microbial, anti-tubercular, and anti-protozoal properties. These compounds have been proven by pharmacological investigations to be efficient against different strains of microbes. Therefore, in this review, the structural variations of BnZ are listed along with various applications, predominantly related to their biological activities.

## 1. Introduction

With regard to BnZ’s limiting efficacy and its extensive advantageous reactive properties for quantitative and qualitative relations, BnZ derivatives have remarkable beneficial components due to their diverse bioactivity and therapeutic uses [1]. Due to the importance of BnZ, it was decided to synthesize a number of novel BnZ-based derivatives with additional heteroatoms and investigate their probable bioactivity. BnZ exhibits a significant electron-rich heterocyclic pharmacophore in its structure which is beneficial for drug design and development [2]. Investigation of the melting point (m.p.) of several BnZ derivatives showed that addition of a substituent to 1-position leads to a reduction in m.p. The presence of two nitrogen atoms in the imide group generally causes polarity, resulting in its solubility in organic solvents and greater solubility in polar solvents. The solubility in non-polar solvents may improve with the addition of non-polar substituents to the BnZ ring in various positions. In contrast, the addition of polar groups to BnZ causes it to become more soluble in polar liquids. Over 300 °C, the BnZ derivatives’ structures are unaltered. BnZ is soluble in dilute acid and has less basicity than imidazole. Additionally, BnZ has sufficient acidity to dissolve in aqueous (aq.) alkali for the synthesis of N-metallic compounds. Similar to imidazole, BnZ’s acidic character shows ion stabilization by resonance. In a weak basic solution such as K_2_CO_3_, BnZ is more acidic with improved solubility in an aqueous medium [3]. Thus, the addition of substituents to the BnZ ring at different positions can significantly impact properties such as melting point and solubility in organic and polar solvents. However, BnZ shows significant electronic transition because of its heterocyclic pharmacophore, which contributes to its pharmacological potential. Furthermore, its unique characteristics enable structural modulation of BnZ and its derivatives, which allows for solubility in dilute acid. Therefore, by exploring novel BnZ-based structural variations with additional heteroatoms, further insights into their bioactivity can be gained. These factors highlight the importance of BnZ derivatives and their potential applications.

## 2. Synthesis of Benzimidazole

**Scheme 1:**
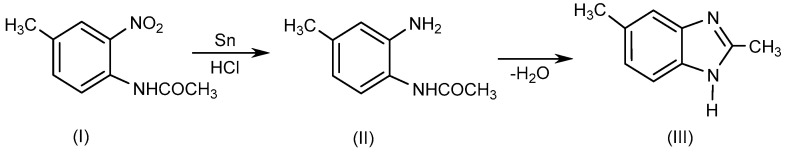
Hoebrecker method.

BnZ is a bicyclic heterocyclic aromatic compound which is made up of benzene and imidazole rings bonded at 4- and 5-positions. Ortho-phenylene derivatives of BnZ such as methyl-o-phenylenediamine are known as benzoglyoxalines. Recently, several researchers have described various techniques for synthesizing 1- or 1, 2-disubstituted BnZ derivatives by employing various moieties in various reaction environments [4]. Initially, BnZ was synthesized as a 2,5-dimethyl-benzimidazole (III) in 1872 by Hoebrecker [5] through the reduction of 2-nitro-4-methylacetanilide (I) using tin (Sn) and hydrochloric acid (HCl), followed by the dehydration of 2-nitro-4-methylacetanilide(II), as explained in Scheme 1. Details regarding the specific reaction conditions are provided elsewhere [6].

**Figure 1:**
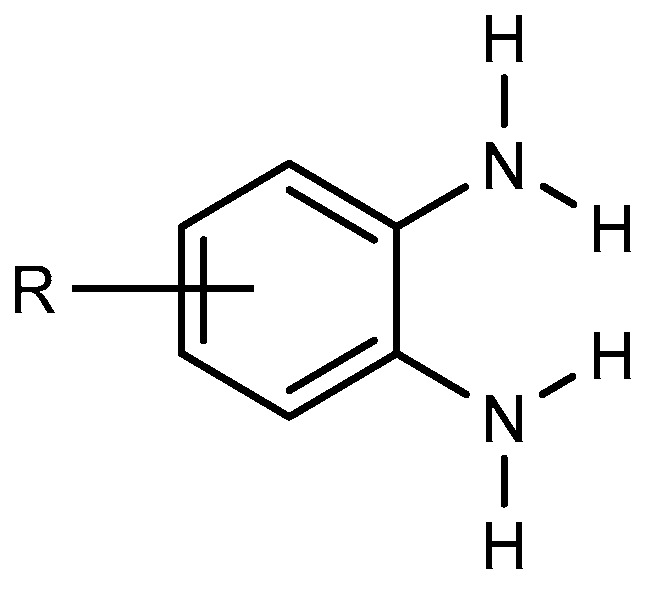
Orthophenylenediamine compound.

BnZ synthesis proceeds by choosing benzene derivatives that have nitrogen-containing functionalities at ortho positions to each other, e.g., orthophenylenediamine (Figure 1). It is evident that the starting material must have the functionality to enable the preparation of BnZ using a variety of techniques. Ortho-phenylenediamine and its derivatives undergo a condensation process with carboxylic acids or aldehydes. Therefore, several BnZ synthesis processes have been categorized based on the basic nature of o-phenylenediamine [7]. The direct condensation of orthophenylenediamine with nitriles has much use in the synthesis of benzimidazoles as well as in the manufacture of benzimidazole-related agricultural and pharmaceuticals chemicals because nitriles are easily accessible due to their broad supply as commodity chemicals [8].

**Scheme 2:**
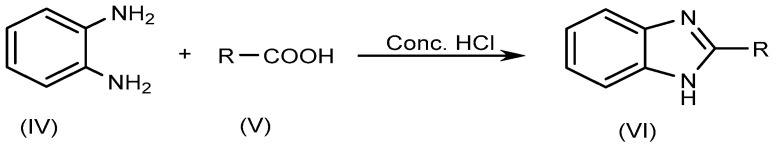
Phillip’s method.

The most typical synthetic approach for producing several BnZ derivatives is known as Phillip’s method, which entails the condensation of o-phenylenediamine (IV) with carboxylic acids (V) itself or its derivatives, and which involves heating the reagents in the presence of concentrated HCl (Scheme 2).

A number of new modified BnZ derivatives have been synthesized, having a furan substituent at the second position and an alkyl/aryl substituent at the first position, which further was tested by an in silico study [9].

**Figure 2:**
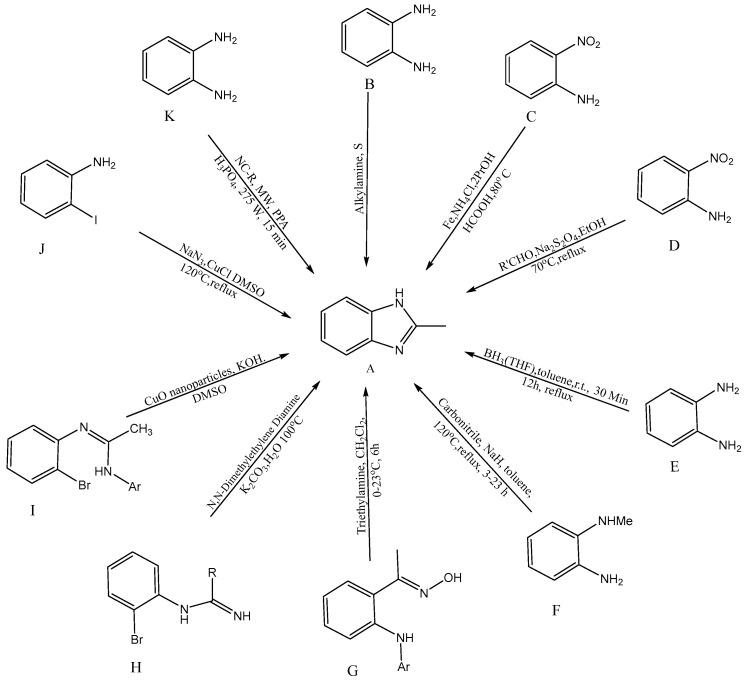
Synthetic approaches for BnZ.

Several synthetic tactics have been successfully implemented, which are described in Figure 2 for the production of BnZ [A]. A combination of ortho-substituted aniline [B] and bifunctional o-esters produced a BnZ derivative in 2012, according to Bastug et al. [10]. Utilizing heterocyclic aromatic compounds like H_2_NC_6_H_4_NO_2_ with CH_2_O_2_, iron powder, and ammonium chloride, proceeded by the one pot reduction of NO_2_ followed by imidazole cyclisation [C] into bicyclic 2-H BnZ, was one method used by Hanan et al. (2010) [11]. Yang et al. developed a new method for the one-step synthesis of 2-substituted -N-H, -N-alkyl, and -N-aryl BnZ derivatives in the presence of aldehydes and sodium dithionite through the reduction of o-nitroanilines [D], succeeding in synthesizing BnZ [12]. Another approach for the synthesis of 2-substituted BnZ was published by Cui et al. in 2012 for producing 1,2-phenylenediamines and triacyloxyborane intermediates, which were produced via the interaction between carboxylic acids [E] and borane-THF, to yield 2-substituted BnZ [13]. N-methyl-1,2-phenylenediamine, sodium hydride, and carbonitriles [F] were used as the starting ingredients by Sluiter and Christoffers in 2009 [14]. Wray synthesized [1] 1H-indazoles from common arylamino oximes in 2010 in the presence of several bases, but the synthesis of BnZ was only improved by one base, i.e., triethylamine [G] [15]. Cuprous oxide, potassium carbonate, (CH_3_NH)_2_C_2_H_4_, and water [H] were used by Peng et al. to synthesize cost-effective and environmentally friendly BnZ [16]. Ortho-bromoaryl compounds are cyclized intramolecularly using cuprous oxide nanoparticles like a catalyst [I]. Saha et al. were able to synthesize substituted BnZ and 2-aminobenzimidazole. The catalyst might also be restored without altering its activity, in addition, with this method [17]. CuI/l-proline was applied by Diao et al. as a catalyst to condense 2-iodoacetanilides and aqueous ammonia for cyclization to create substituted 1H-BnZ by applying heat under acidic circumstances [18]. Kim et al. produced BnZ derivatives by condensation as well as the formation of C-N bonds. Sodium azide, RCHO [J] and 2-haloanilines are active as initial ingredients in one-pot synthesis [19]. Tao Zhang initially presented a model for the reaction of BnZ with [K] (10 mmol) and benzonitrile (NC-R) in a mixture of 20 mL PPA (polyphosphoric acid) and 10 mL H_3_PO_4_ under microwave irradiation (MC 275 W, 15 min), with a yield of 92% [20].

Several synthetic tactics have been successfully implemented, which are described in Figure 2 for the production of BnZ [A]. A combination of ortho-substituted aniline [B] and bifunctional o-esters produced a BnZ derivative in 2012, according to Bastug et al. [10]. Utilizing heterocyclic aromatic compounds like H_2_NC_6_H_4_NO_2_ with CH_2_O_2_, iron powder, and ammonium chloride, proceeded by the one pot reduction of NO_2_ followed by imidazole cyclisation [C] into bicyclic 2-H BnZ, was one method used by Hanan et al. (2010) [11]. Yang et al. developed a new method for the one-step synthesis of 2-substituted -N-H, -N-alkyl, and -N-aryl BnZ derivatives in the presence of aldehydes and sodium dithionite through the reduction of o-nitroanilines [D], succeeding in synthesizing BnZ [12]. Another approach for the synthesis of 2-substituted BnZ was published by Cui et al. in 2012 for producing 1,2-phenylenediamines and triacyloxyborane intermediates, which were produced via the interaction between carboxylic acids [E] and borane-THF, to yield 2-substituted BnZ [13]. N-methyl-1,2-phenylenediamine, sodium hydride, and carbonitriles [F] were used as the starting ingredients by Sluiter and Christoffers in 2009 [14]. Wray synthesized [1] 1H-indazoles from common arylamino oximes in 2010 in the presence of several bases, but the synthesis of BnZ was only improved by one base, i.e., triethylamine [G] [15]. Cuprous oxide, potassium carbonate, (CH_3_NH)_2_C_2_H_4_, and water [H] were used by Peng et al. to synthesize cost-effective and environmentally friendly BnZ [16]. Ortho-bromoaryl compounds are cyclized intramolecularly using cuprous oxide nanoparticles like a catalyst [I]. Saha et al. were able to synthesize substituted BnZ and 2-aminobenzimidazole. The catalyst might also be restored without altering its activity, in addition, with this method [17]. CuI/l-proline was applied by Diao et al. as a catalyst to condense 2-iodoacetanilides and aqueous ammonia for cyclization to create substituted 1H-BnZ by applying heat under acidic circumstances [18]. Kim et al. produced BnZ derivatives by condensation as well as the formation of C-N bonds. Sodium azide, RCHO [J] and 2-haloanilines are active as initial ingredients in one-pot synthesis [19]. Tao Zhang initially presented a model for the reaction of BnZ with [K] (10 mmol) and benzonitrile (NC-R) in a mixture of 20 mL PPA (polyphosphoric acid) and 10 mL H_3_PO_4_ under microwave irradiation (MC 275 W, 15 min), with a yield of 92% [20].

## 3. Metal-Catalyzed Derivatives of Benzimidazole

**Scheme 3:**
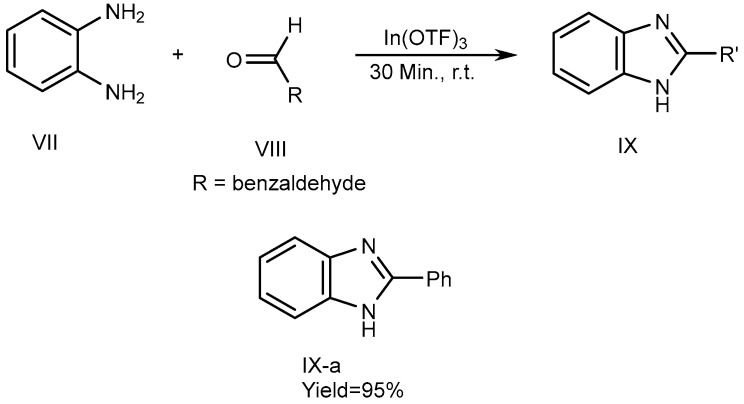
Metal-catalyzed reduction of BnZ.

Metal catalysts have a few features, including the fact that they can be recovered. Catalysts have surface efficiency for catalyzing reactions and further increase overall reaction rate. Indium [III]triflate was used as a reusable catalyst by De et al., in 2006 which described the synthesis of MSBs (IX-a) with high yields. The synthesis of 2-substituted BnZ without solvent occurs by the fusion of o-phenylenediamine (VII) with aldehydes [where R = benzaldehyde (VIII)] utilizing a catalytic quantity of In(OTf)_3_. VII and VIII were combined in a 1:1.1 molar ratio and heated up to atmospheric temperature for 30 minutes to perform the reactions. They also used various catalysts, such as Copper [II]triflate, Lutetium[III]trifluoromethanesulfonate, Indium[III]trifluoromethanesulfonate and La(OTf)_3._ Among these, the most recently used catalysts gave the highest output. The process is depicted in Scheme 3 [21].

**Scheme 4:**
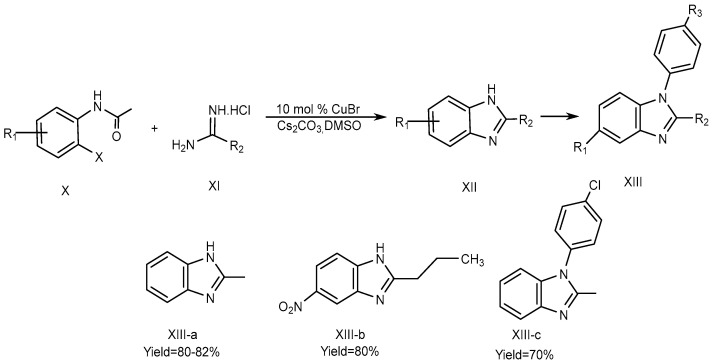
CuBr-catalyzed reactions.

Fu et al., described the method for the synthesis of 2-substituted 1H-BnZ (XIII a-b) in dimethyl sulfoxide using cesium carbonate as a base and 10 mol % Copper[I] bromide as a catalyst. The optimized yield of 2-substituted 1H-BnZ (XIII-c) using this approach was obtained by coupling, hydrolysis, and the intra-molecular cyclization of o-haloacetanilide (X) derivatives with amidines (XI). o-iodoacetanilide is required for the N-arylation of amidines at 60 °C to yield XIII-a, whereas o-bromoacetanilide required a high temperature of 90 °C to yield XIII-b and XIII-c., which is displayed in Scheme 4 [22].

**Scheme 5:**
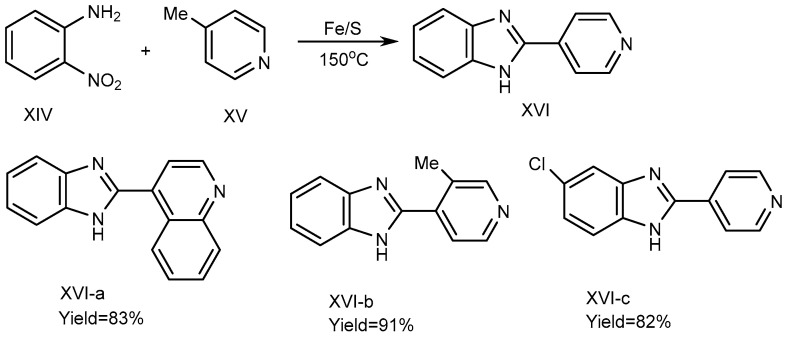
Fe/S-catalyzed reaction of BnZ.

In 2013, Nguyen et al. described a process using iron-sulfur as a catalyst to synthesize the BnZ derivative (XVI). For the synthesis of XVI, 4-picoline (XV) and substituted o-nitroanilines (XIV) were used as reactants, and the reaction was carried out at 150 °C under solvent-free conditions using an equimolecular amount of an Fe/S catalyst. Fe/S played a crucial role for promoting the cyclization process. Pyridine and quinoline derivatives possess a methyl group at the 2- or 4-position (XV) and are coupled with XIV to form derivative MSBs (XVI a-c), where all other suitable substrates in the reaction along with o-nitroaniline synthesize the corresponding benzimidazole products XVI-a-b with yields of 83-91%, if o-Nitroanilines have electron-donating and withdrawing substituents on its benzene ring. There are methyl groups of picolines in elemental Chloride (XVI-c), as depicted in Scheme 5 [23].

**Scheme 6:**
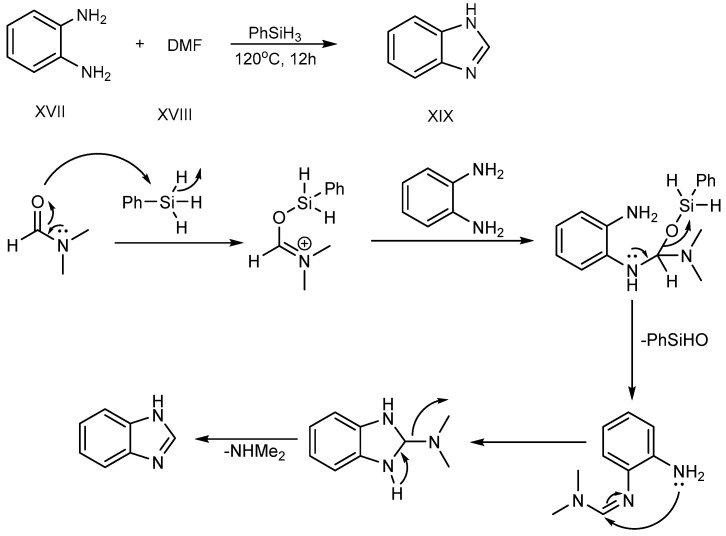
Cyclization of o-phenylenediamines with DMF.

Sun et al. (2017) described a procedure for the feasible condensation of phenylhydrosilicon, o-phenylenediamine (XVII) and dimethylformamide (XVIII). According to this scheme, hydrosilicon is utilized as a reaction catalyst, activating the carbonyl group of di-methylformamide to make an Si–O bond at 120 °C for 12 hours. An amine-group in o-phenylenediamine was coupled with activated DMF-hydrosilicon mixture, and further undergoes cyclization, succeeding in the excellent synthesis of BnZ (XIX), as shown in Scheme 6 [24].

**Scheme 7:**
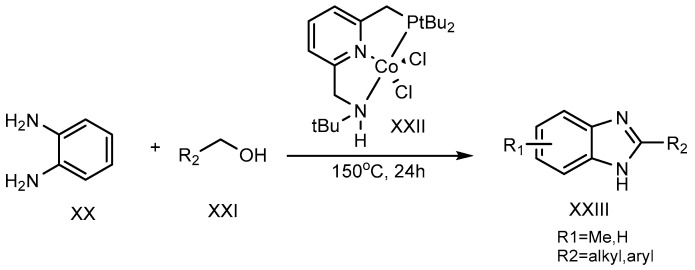
Co-complex catalyst-mediated reaction.

A dehydrogenation reaction, using cobalt-pincer complexes (XXII) as a catalyst, was carried out at 150 °C for 24 hours by Milstein et al. (2017). Primary alcohol (XXI) and o-phenylenediamine (XX) were combined to synthesize the 2-MSB derivative (XXIII) (Scheme 7) [25].

## 4. Metal-Free Catalyzed Derivatives of BnZ

**Scheme 8:**
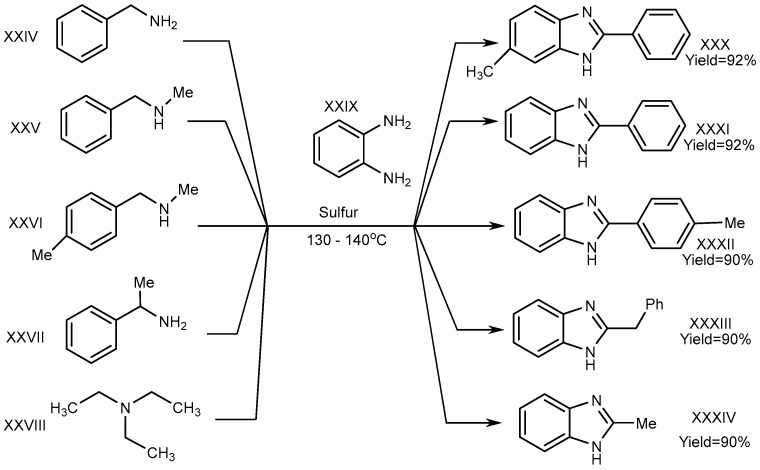
Cross–coupling reaction of o–phenylenediamines with aliphatic amines (S).

A one-pot synthesis with an exceptional yield was reported by Nguyen et al. in 2012 under dry and metal-free conditions to obtain XXX–XXXIV. In this method, the reaction was completed with trialkyl amine (triethylamine) (XXVIII), benzene-1,2-diamine (XXIX), and derivatives of phenylmethanamines (XXIV–XXVII). Sulphur atoms helped to proceed the reaction for generating C–N bonds, and the researchers succeeded in its cyclization (Scheme 8) [26].

**Scheme 9:**
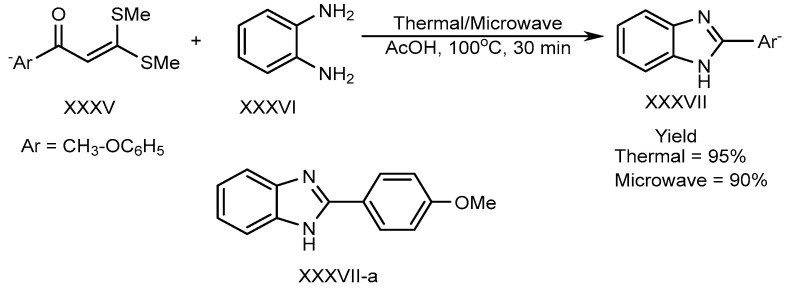
Thermal/microwave-assisted BnZ synthesis.

Shanmugam et al. described the efficient metal-free synthesis of aryl-substituted BnZ (XXXVII-a) through the use of acetic acid as a catalyst under microwave heating conditions. This synthesis involves the reaction of o-phenylenediamine (XXXVI) with a substituted aromatic aldehyde (XXXV). Shown in Scheme 9, the reaction is entirely free from metals, as well as using environmentally friendly green solvent [27].

**Scheme 10:**
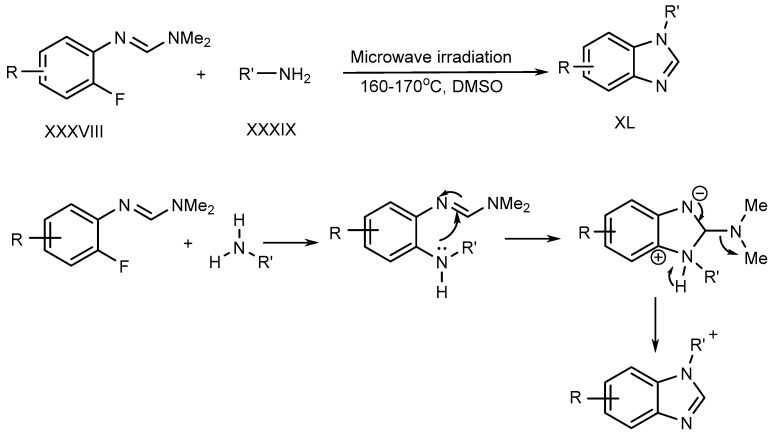
Synthesis of BnZ under metal-free conditions with amines.

Liu et al. described a metal–free process for the preparation of N-substituted BnZ (XL) under microwave irradiation. The improved yield is produced when fluoro-aryl formamidines (XXXVIII) with primary amines (XXXIX) undergo an S_N–_Ar reaction [28]. (Scheme 10). 

**Scheme 11:**
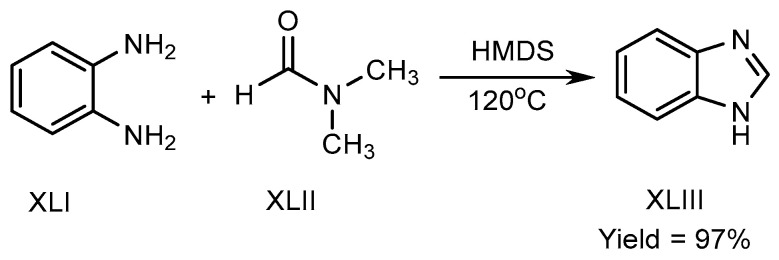
Metal-free BnZ biosynthesis.

Mostafavi et al. published a method in 2018 for BnZ biosynthesis. Hexamethyldisilazane regulated the reaction at 120 °C, and transformed o-phenylenediamine (XLI) and N,N-Dimethylformamide into BnZ. There is no need for any acid, transition metal, or fluid for proceeding the reaction to achieve good yields XLIII (Scheme 11) [29].

**Scheme 12:**
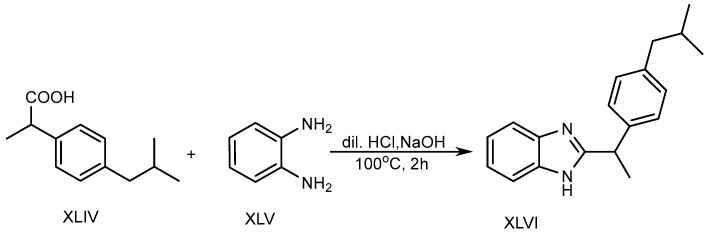
Base-catalyzed synthesis of BnZ.

In the presence of 5ml of dilute hydrochloric acid solution, o-phenylenediamine (XLV) and Ibuprofen (XLIV) are used to produce a BnZ variant XLVI. Further, the mixture was heated in a water bath at 100 °C for two hours with the slow addition of 10% sodium hydroxide, as shown in Scheme 12. The researchers discovered that BnZs have very strong antimicrobial properties [30].

## 5. Green Synthesis of Benzimidazole

Most chemical organizations or industries, including pharma-enterprises, are now coping with mild ecological issues, like excessive solvent, chemicals waste, and the use of catalysts in the production of BnZ. Green synthesis mediates solvent-free production by using environmentally friendly catalysts like C_3_H_6_O_3_ or B(OH)_3_ under microwave condition, which is an effective way to get rid of these issues.

**Scheme 13:**
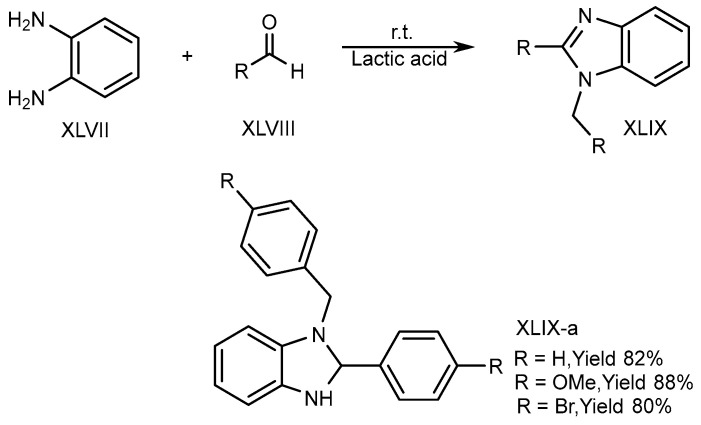
Synthesis of XLIX-a in green solvent.

XLVII and aldehydes (XLVIII) undergo cyclic condensation in an equal amount of a bioabsorbable solution like ethanol and lactic acid for 4-6 hours at normal temperature. Song et al. (2016) disclosed the one-pot synthesis of disubstituted-benzimidazole (DSBs) (XLIX-a) derivatives. The optimized result is obtained in lactic acid (Scheme 13) [31].

**Scheme 14:**
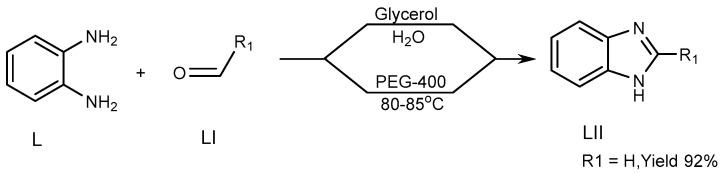
Synthesis of BnZ in solution instead of PEG.

Khunt et al. (2014) described a strategy for the preparation of a BnZ candidate (LII) via the interaction of 1,2-diaminobenzene (L) with aldehydes (LI) by use of an ecofriendly solvent such as polyethylene glycol (PEG)-400. Above 80–85 °C, the process produces the highest yield of PEG-400. The targeted molecule was produced through a new unique synthetic pathway, in which glycerol/water is used (Scheme 14) [32].

**Scheme 15:**
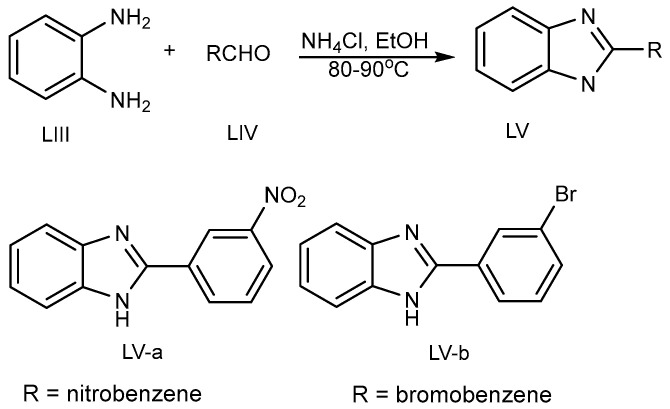
BnZ synthesis using ammonium chloride.

Kathirvelan et al. discovered the one-pot synthesis of a monosubstituted benzimidazole (MSB) (LV a-b) scaffold in 2013 (Scheme 15) by the coupling of benzene-1,2-diamine (LIII) and aldehydes (LIV) using an ecofriendly feasible catalyst, i.e., ammonium chloride in ethanol at 80–90 °C [33].

**Scheme 16:**
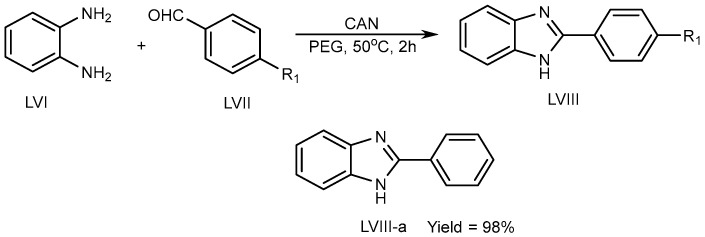
CAN-catalyzed polyethylene glycol–MSB reaction.

Kidwai et al. (2010) described a unique approach using ceric ammonium nitrate (CAN) catalyst for the synthesis of BnZ scaffold (LVIII) in a polyethylene glycol at 50 °C for 2 h. Benzene-1,2-diamine (LVI) combined with aldehyde (LVII) in CAN catalyst tends to lead to exceptional PEG yields (LVIII-a) (Scheme 16) [34].

**Scheme 17:**
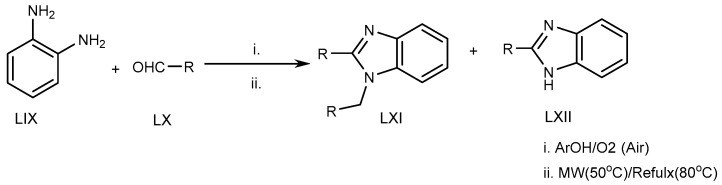
Microwave-irradiation-mediated BnZ synthesis.

Azarifar et al. (2010) suggested an eco-friendly microwave-assisted reflux reaction of o-phenylenediamine (LIX) with an RCHO derivative (LX) at 80 °C for the synthesis of MSBs and DSBs (LXI, LXII) (Scheme 17) [35].

## 6. Multifaceted Efficacy of Benzimidazole

**Figure 3:**
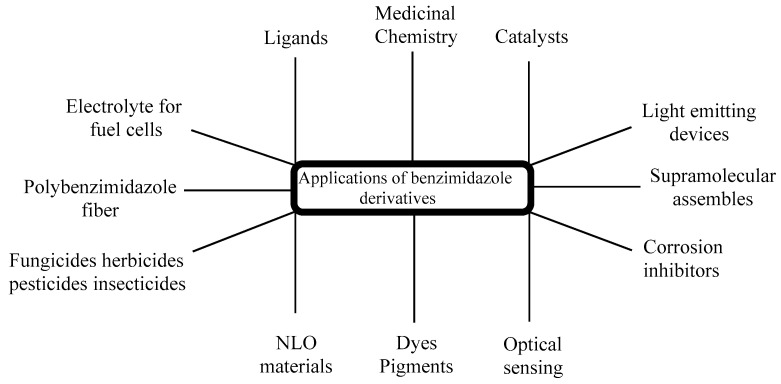
Applications of BnZ derivatives.

The significance of chiral BnZs has led to a relatively new sector of drug discovery for various biological applications; therefore, its synthesis has received special attention [36]. Additionally, chiral BnZs have been utilized as organocatalysts for the enantioselective chlorination, Diels–Alder reactions, asymmetric Michael additions, and asymmetric aldol-type reactions. However, Rh- and Pd-based BnZ complexes are synthesized using a Mizoroki–Heck reaction [37]. The reduction processes is performed using Suzuki–Miyaura coupling reactions [38]. Earlier surveys demonstrate that the BnZ scaffold is crucial for its therapeutic usage, in addition to as optical sensors for use in nanomaterial properties [39]. These properties can apparently benefit the numerous sensing devices, as well as its cost effectiveness and operational flexibility; therefore, it seems to have specific usage in medicine, geology, and industrial innovation (Figure 3). Another use of BnZ and its derivatives is in supra-molecular assemblies with intriguing features for various uses, such as thermostable polymers, adsorbent materials, nano-containers, and liquid crystals for electronic conduction. For the last few years, it has been seen in a lot of research in the area of polybenzimidazole (PBI) derivatives, such as for solid electrolytes of fuel cells [40,41,42,43,44], fiber [45], thin coatings [46], advanced protective coverings [47], or to remove palladium, Th, and U from a watery medium [48]. PBI-based technologies exist in Charlotte, North Carolina, used in the pioneering industrial applications for firefighter safety in Europe, United States, and Middle East, with 32 years of expertise. PBI materials are famous for their tested protection against heat and flames, and shield the firemen in a various fire services [49].

Another use of poly-Benzimidazole, as a combined matrix, shows extraordinarily strong evaporation property due to its high porosity and selectivity [50]. Additionally, this is the most recommended material for another photonic technology in organic frames. This has led to the study of a number of small molecules of BnZ derivatives like 2-mercaptobenzimidazole, 2-phenylbenzimidazole, and 2-hydroxybenzimidazole, with excellent non-linear optical (NLO) properties [51] and highly intricate structures [52]. Benlate and Carbendazim both used BnZ as fungicides with minimal cytotoxicity at lower concentrations and no risk for carcinogenic or genetic effects [53]. There is documentation regarding the utilization of BnZ as fertilizers and insecticides, which also specifies their uses [54]. BnZ is an organic molecule, recently highlighted as a corrosion inhibitor for copper, iron, or zinc in acidic circumstances [55,56] because of its properties of BnZ-bonded electron donating and accepting substituents. Scientists have demonstrated that BnZ is a flexible and necessary chromophore for natural dyes, with excellent photophysical, electrochemical, and photovoltaic characteristics [57]. Benzimidazol-2-one is highly considerable because of its durability and light-resistance properties. It has been used for the last three decades to produce a wide variety of subtleties in water for color painting and electro-photographic toner [58]. BnZ has proven to be a vital molecule in organic light-emitting devices (OLEDs), with excellent phosphorescence, thermal features, and morphological stabilities [59]. For innovative research with a new mode of action, heterocyclic compounds are frequently used. Aryl aziridines is a heterocyclic compound [60] and BnZ derivatives play a significant role because of the vast range of biological actions they exhibit. According to a literature survey, BnZ and its derivatives have physiological and pharmacological activities. These are able to treat a broad range of disorders, particularly epilepsy, diabetes, and pregnancy. Such derivatives exhibit a variety of bioactivities like antiallergic [61], antihistamine [62], anti-ulcerative, antiproliferative [63], antioxidant [64,65], antifungal, antibacterial, antitubercular [66], antihypertensive [67], anti-inflammatory, analgesic, antiamoebic [68], antitumor [69,70,71], antimalarial [72], and anti-kinase activity [73]. Several BnZ derivatives are also being investigated as cholinesterase inhibitors and anti-parasitic drugs [74,75,76,77].

## 7. Benzimidazole-Derivative-Based Biological Activities

**Figure 4:**
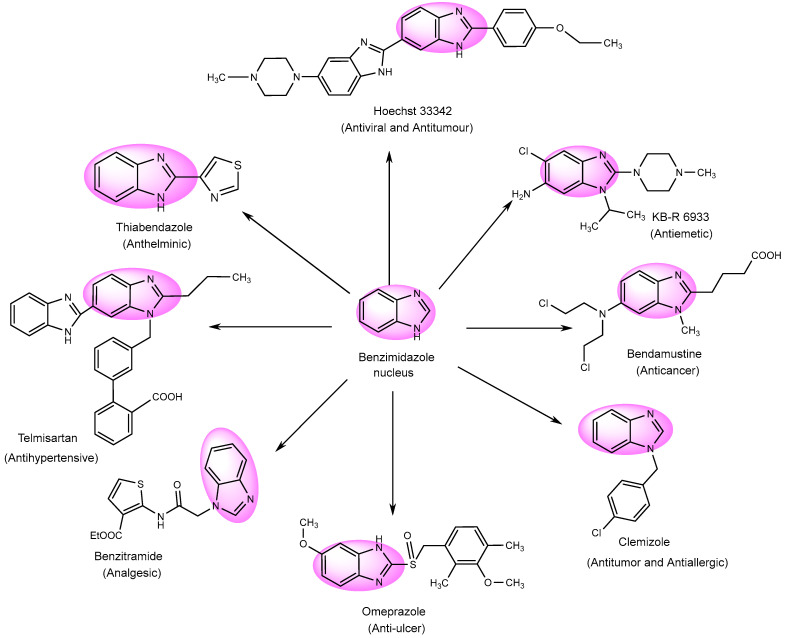
Multifunctional examples of BnZ derivatives.

Numerous derivatives have been explored in individual investigations, for various therapeutic treatments. These include the antiemetic, KB-R-6933; the anti-cancer, Bendastumide; the antiviral, l Hoechst 33342; the antihistamine, Clemizole; the anti-ulcerative, Omeprazole; and the anti-hypertensive, Telmisartan. Additionally, Thiabendazol, an anti-fungal agent, has been employed to examine its effects (Figure 4) [78].

## 8. Anti-Cancer Activity

Cancer is responsible for the second-largest number of disease-related deaths in the world. Anticancer drugs must harm cancer-affected cells but not ordinary cells, and act effectively against cancer. For the last few years, anti-cancer medicines have shown relatively significant damage to both normal and tumor cells. Cancer patients are discontinuing chemotherapy because of toxicities and the side effects of anticancer medicines. It should be observed that the investigation of novel anticancer medicines with a high efficacy and minimal side effects are both an urgent necessity and a challenging problem.

**Figure 5:**
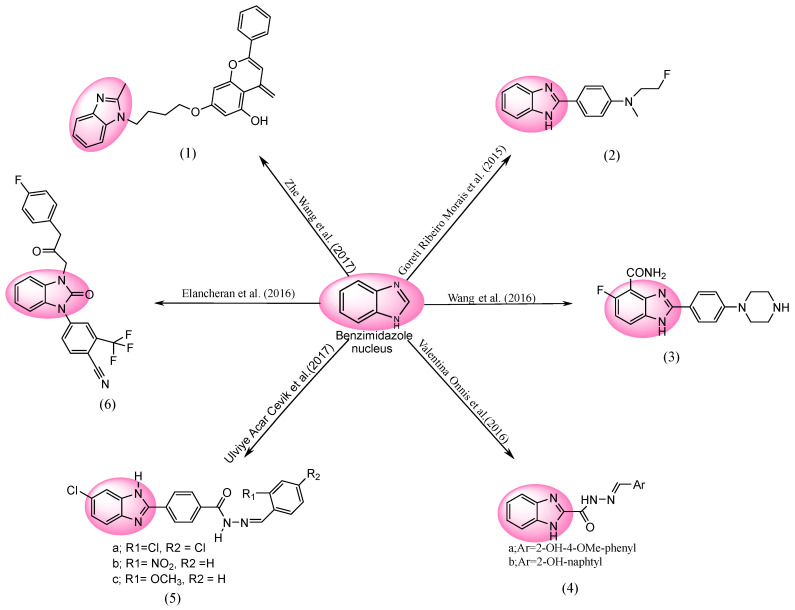
BnZ-based anti-cancer drugs [79,80,81,82,83,84,85].

In Figure 5, Zhe Wang et al. evaluated the anti-cancer activities of several chrysin-based BnZ derivatives. Entity 1 (7-(4-(2-methyl-1H-benzo[d]imidazol-1-yl)butoxy)-4-methylene-2-phenyl-4H-chromen-5-ol) was synthesized and exhibited the most anti-proliferative effects on MCF cell lines, having an IC_50_ of 25.72 ± 3.95 μM. Flow cytometry results showed that drug 1 accelerates MCF cell-line apoptosis in a daily dosage manner. When the anti-cancer activity of compound 1 was examined in tumor-suffering mice, the resultant tumor growth was shown to be suppressed [79]. Goreti Ribeiro Morais et al. [80] freshly developed BnZ variants with Na_3_AlF_6_ and R-OH substituents, which has been further tested for anti-cancer activity. “Drug 2” (4-(1H-benzo[d]imidazol-2-yl)-N-(2-fluoroethyl)-N-methylaniline) was demonstrated to be the most significant anticancer drug. The BnZ nucleus was substituted by a 2-fluoroethyl string at the aniline nitrogen, which demonstrated acceptable cytotoxicity against U87 glioblastoma cell lines (IC_50_ = 45.2 ± 13.0 μM) compared to DOX (IC_50_ = 16.6 ± 2.5 μM), i.e., a generic anticancer medicine [81].

Unique 5-fluoro-benzimidazole-4-carboxamide analogues were synthesized by Wang et al. One highly effective anti-cancerous drug, 3 (5-fluoro-2-(4-(piperazin-1-yl)phenyl)-1H-benzo[d]imidazole-4-carboxamide), was identified to have an IC_50_ of 7.4 μM, with good cell inhibition against HCT116 cell lines [82]. The antiproliferative effect of BnZ derivatives was investigated by Valentina Onnis et al. All investigated cell lines were precisely suppressed in terms of tumor growth by the hydrazones 4a ((E)-N′-(2-hydroxy-4-methoxybenzylidene)-1H-benzo[d]imidazole-2-carbohydrazide) and 4b ((E)-N1-((2-Hydroxynaphthalen-1-yl)methylene)-1H-benzo[d]imidazole-2-carbohydrazide). A growth inhibitor was tested against cell lines of human T-lymphoblastic leukemia for entity 4a and 4b, with an IC_50_ of 0.98 ± 0.02 and 1.0 ± 0.01 μM, and human pancreatic carcinoma, with an IC_50_ of 6.3 ± 3.2 and 7.9 ± 0.3 (μM), and murine leukemia, with an IC_50_ of 1.6 ± 0.9 and 2.9 ± 1.3 μM, respectively. They noticed that mixing BnZ with hydrazone enhanced its pharmacological activities, producing a new featured chemical compound that has strong antiproliferative properties [83]. Recently, the anti-cancer effect of BnZ derivatives was investigated by Ulviye Acar Cevik et al. Besides these examined chemicals, they also noticed that compounds 5a (4-(6-chloro-1H-benzo[d]imidazol-2-yl)-N′-(2,4-dichlorobenzylidene)benzohydrazide) and 5b (4-(6-chloro-1H-benzo[d]imidazol-2-yl)-N′-(4-nitrobenzylidene)benzohydrazide) had noticeable cytotoxicity. Compounds 5a and 5b, when applied to human-lung-cell-line adenocarcinoma, have an IC_50_ of 0.0316 μM. Compared with the classical anticancer medicine cisplatin (IC_50_ 0.045 and 0.052 μM), 5c (4-(6-chloro-1H-benzo[d]imidazol-2-yl)-N′-(2-methoxybenzylidene)benzohydrazide) showed significant cytotoxicity, with an IC_50_ of around 0.06 μM [84]. Elancheran et al. synthesized and examined a number of oxo-bBnZ derivatives for their ability to inhibit androgen receptor functions. They had effective actions against PC-3, with an IC_50_ of 12.19 ± 0.25 μM, and on LNCaP, with an IC_50_ of 11.2 ± 0.13 μM. Also, cytotoxicity effects were actively expressed by compound 6 (4-(3-(3-(4-fluorophenyl)-2-oxopropyl)-2-oxo-2,3-dihydro-1H-benzo[d]imidazol-1-yl)-2-(trifluoromethyl)benzonitrile) [85].

## 9. Anti-Inflammatory and Analgesic Activity

**Figure 6:**
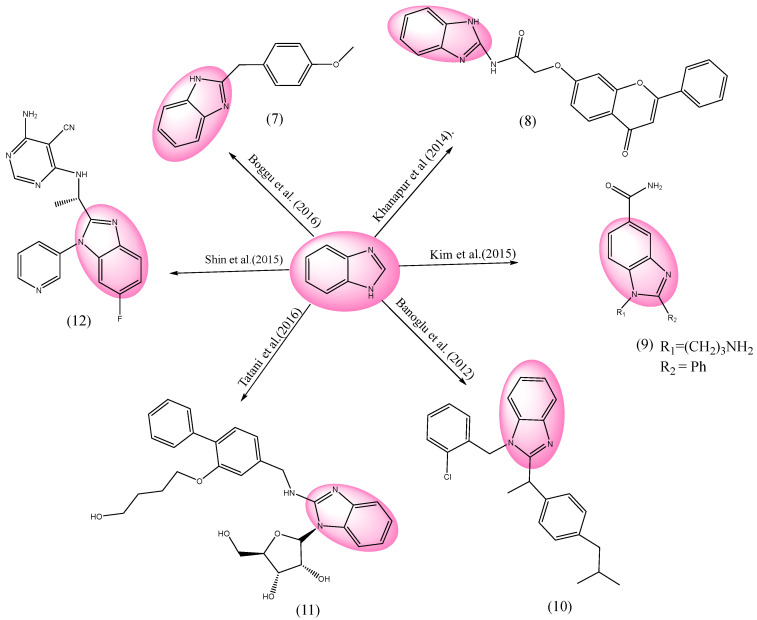
BnZ-based anti-inflammatory drugs [89,90,91,92,93,94].

Inflammation is an important characteristic of the body’s immunological system. The common early symptoms and indications are heat, redness, pain, swelling, and the lack of ability of the immune system. Since non-steroidal anti-inflammatory drugs (NSAIDs) naturally have the capacity to block cyclooxygenases (COXs), they are frequently employed to reduce inflammation. The synthesis of prostaglandins from arachidonic acid is mediated by cyclooxygenase. Medical scientists have investigated a number of analgesic and anti-inflammatory drugs using BnZ bases [86,87,88], as shown in Figure 6.

One of the BnZ-derivative compounds 7 (2-(4-methoxybenzyl)-1H-benzo[d]imidazole) was investigated by Boggu et al., and had the greatest nuclear factor (κB) at its optimized concentration, with an IC_50_ of 1.7 μM. An additional synthesis for the possible suppression of activity was examined in unsubstituted BnZ, which had only one carbon spacer and a methoxy group towards the phenyl ring. By refluxing 2-aminobenzenethiol using xylene in the presences of 4-methoxyphenylacetic acid, white solid benzothiazole with a yield of 48% and a melting point 60–62 °C was produced [89]. Khanapur et al. synthesized BnZ derivatives, and examined their effect. Remarkable anti-inflammatory effect was shown by compound 8 (N-(1H-benzo[d]imidazol-2-yl)-2-((4-oxo-2-phenyl-4H-chromen-7-yl)oxy)acetamide). Several heterocyclic moieties were bonded with an acetamide that enhanced their activities, with substituted ester at the 7th position. It was revealed that bicyclic moieties are more effective with substituents at the five and six position throughout heterocyclic molecules. Cyclooxygenase (COX) inhibition at 10 μM for COX 2 was 85.91 ± 0.23%, with an IC_50_ of 3.11 ± 0.41 μM, and for COX 1 was 25.91 ± 0.77%, with an IC_50_ of 57.89 ± 0.19 μM. Using column chromatography (petroleum ether/EtOAc), the residue was purified as a colorless solid with a yield of 87% [90]. Only mild inhibitory action was demonstrated by the BnZ derivative 9 (2-(2-aminoethyl)-1-(3-aminopropyl)-1*H*-benzo[d]imidazole-5-carboxamide), which was synthesized by Kim et al., who chose a 3-aminopropyl group as the substituent at the N1 antagonist to Janus kinase 1 (JAK1), with an IC_50_ of 6.1 and 11.3 μM, and Janus kinase 3 (JAK3), with an IC_50_ of 1.5 and 1.8 μM [91]. Entity 10 (1-(2-chlorobenzyl)-2-(1-(4-isobutylphenyl)ethyl)-1H-benzo[d]imidazole) is a collection of BnZ derivatives synthesized by Banoglu et al., with an isobutyl-phenylethyl profile of ibuprofen, which noticeably reduced cellular 5-Lipoxygenase (5-LO), with an IC_50_ value of 0.31 μM, but was considerably less effective on free-cell 5-LO, which was hardly inhibited by 23% at 10 μM [92]. However, its inhibitory effects towards human concentrative nucleoside transporter 2 (hCNT2) and rat concentrative nucleoside transporter 2 (rCNT2) was greatly improved after substituting adenine, upon which the resultant BnZ product 11 ((2S,3R,4S,5R)-2-(2-(((2-(4-hydroxybutoxy)-[1,1′-biphenyl]-4-yl)methyl)amino)-1H-benzo[d]imidazol-1-yl)-5-(hydroxymethyl)tetrahydrofuran-3,4-diol) was formed. So, the most effective hCNT2 inhibitor examined was the semi-synthetic derivative of BnZ 11, with an IC_50_ value of 0.062 μM. This was also effective as an rCNT2 inhibitor, with an IC_50_ of 1.5 μM. Similarly, compound 11 had a substantially greater solubility of 0.120 mg/mL than others compound in Japanese Pharmacopoeia second fluid (JP2) at 37 °C. Compound 11, in combination with purine, greatly reduced the exaggerated uric acid in plasma at a lower dose of 1.0 mg/kg, which likely seemed to be an in vitro improvement. This compound, at a greater dose (10 mg/kg), did not significantly improve the suppressive effect [93]. Shin et al. assessed the PI3Kd-specific inhibition activity of BnZ derivatives. Substance 12((S)-4-amino-6-((1-(6-fluoro-1-(pyridin-3-yl)-1H-benzo[d]imidazol-2-yl)ethyl)amino)pyrimidine-5-carbonitrile) showed strong PI3Kd activity. At day 10, plasma was collected after examining exposures of different doses of compound 12. Unbound substances were determined and shown via LC-MS/MS with respect to human blood. Unbound phospho-AKT (pAKT) had an IC_50_ of 1.0 μM, phosphoinositide 3-kinase delta (PI3Kδ) had an IC_50_ of 3.1 μM in an in vitro study in mice, and PI3Kβ showed an IC_50_ of 650 μM in an in vitro study in humans. A pyridine ring was substituted by the phenyl moiety, which produced high oral bioavailability [94].

## 10. Anti-Microbial Activity

**Figure 7:**
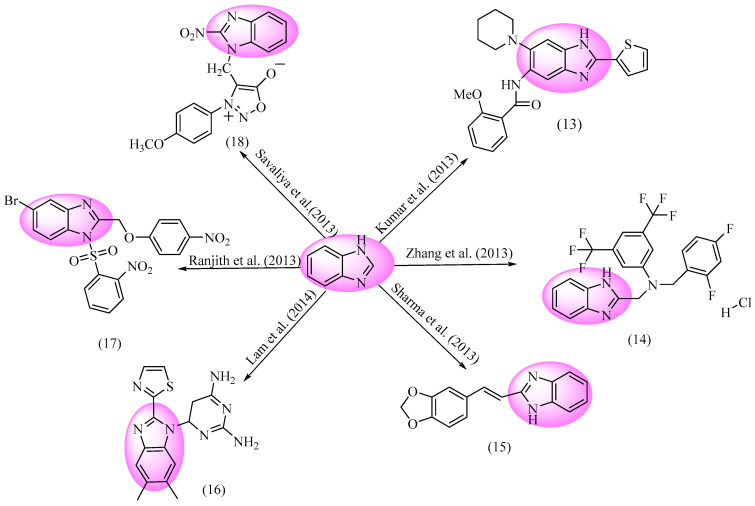
BnZ-based anti-Microbial [99,100,101,102,103,104].

Since they are frequently exploited against microbial infections, certain bacteria have developed resistance to conventional antibiotics. A variety of antibacterial compounds are synthesized to treat and cure microbial illnesses [95]. The flexible and most primary compound is known as a BnZ, which has a wide spectrum of biological actions, most especially for anti-bacterial [96] and antifungal [97,98] properties. Figure 7 depicts BnZ-containing anti-microbial compounds. 

Several BnZ-based variants were developed by Kumar et al., with potential efficiency in antibacterial properties. Entity 13 (2-methoxy-N-(6-(piperidin-1-yl)-2-(thiophen-2-yl)-1H-benzo[d]imidazol-5-yl)benzamide) was evaluated and shown to provide excellent anti-microbial activity. Select compounds have also been assessed for their ex vivo high potency against *F. tularensis* Live vaccine strain (LVS). An ex vivo study of the leading drug 13 against the *F. tularensis* LVS of RAW macrophages was performed. In addition to 13, the dose-dependent leading compound significantly decreased the extent of bacterial action at concentrations of 10 and 50 g/mL. Its MIC_90_ (μg/mL) against *F. tularensis* LVS was 1.6 and its cytotoxicity (μg/mL) on Vero cells was >200 µg/mL with compound 13. Entity 13 is a white solid with a melting point 165–167 °C [99]. Zhang et al. produced variants of BnZ and examined its capability to fight against bacteria. Further, the most effective component was discovered to be 14 (N-((1H-benzo[d]imidazol-2-yl)methyl)-N-(2,4-difluorobenzyl)-3,5-bis(trifluoromethyl)aniline). This had the strongest antibacterial effect against the relevant microorganisms at a dose range from 8–32 mg/mL. Particularly, it had an MIC of 8 mg/mL against the *S. aureus, B. subtilis,* and M. luteus species, as well as 32 mg/mL against *E. coli*. Component 14 has a yellow solid color with a melting point of 137–138 °C [100]. Sharma et al. developed a number of BnZ and benzothiazole equivalents and tested their effectiveness against microorganisms. Resultantly, compound 15 ((E)-2-(2-(benzo[d][1,3]dioxol-5-yl)vinyl)-1H-benzo[d]imidazole) demonstrated excellent efficacy against. *P. aeruginosa,* having corresponding MIC values of 0.01 mM and 3.78 mM. In order to produce novel Schiff bases with heterogeneous complexes using microwaves, methylenedioxy cinnamaldehyde was used as a raw material. Structure activity relationship (SAR) investigations revealed that it increased a broad range of activities compared to the species under study [101]. Lam et al. synthesized several 7-(benzimidazol-1-yl)-2,4-diamino-quinazoline derivatives, and their capability for antibacterial action was assessed. Further, a new investigation of drug 16 (6-(5,6-dimethyl-2-(thiazol-2-yl)-1H-benzo[d]imidazol-1-yl)-5,6-dihydropyrimidine-2,4-diamine) showed it to be the most effective against *S. aureus*, with an MIC of 0.015 mg/mL. A discovered BnZ variant with a thiazol-2-yl group at the 2-position proved to be an incredibly efficient and specific drug. Great potency and selectivity were both exhibited by a thiazol-2-yl group in the BnZ against *S. aureus* dihydrofolate reductase DHFR Ki = 0.002 μM. Compound 16 is white solid in its physical state [102]. Compound 17 (5-bromo-2-((4-nitrophenoxy)methyl)-1-((2-nitrophenyl)sulfonyl)-1H-benzo[d]imidazole) had quite good efficacy against the tested fungi and bacteria. This included notable efficacy against the *M. tuberculosis* H37Rv strain. Additionally, substance 17 was found to be effective against the M. tuberculosis H37Rv strain at around 0.625 μg/mL, but was comparably less efficient against *M. smegmatis,* requiring a concentration of 1.25 μg/mL (ATCC 19420). It is notable that the drug had either an improved or consistent action against *M. fortuitum* with reference to the drug isoniazid (ATCC 19542) at 6.25 μg/mL. It has also shown promising efficacy against the MDR-TB strain. Inhibitory action of Drug 17 against *S. aureus and K. pneumonia* was demonstrated at an MIC ratio of 4 μg/mL. However, its antibacterial potential has already been shown, so these are only the preliminary screening data. More research has been performed to determine the action mechanism [103]. Compound 18 (3-(4-methoxyphenyl)-4-((2-nitro-1H-benzo[d]imidazol-1-yl)methyl)-1,2,3-oxadiazol-3-ium-5-olate) is a different class of BnZ derivatives and was synthesized by Savaliya et al. They discovered its exceptional effectiveness of inhibitory action against Gram-positive bacteria *S. aureus,* with an MIC of 40 μg/mL and inhibition zone (IZ) of 21 μg/mL. Furthermore, its inhibitory action against *B. subtilis,* with an MIC value of 300 μg/mL and an IZ of 12 μg/mL, was noted. It also demonstrated inhibitory action against Gram-negative bacteria *E. coli,* with an MIC value 200 μg/mL and IZ of 14 μg/mL, and against *P. aeruginosa,* with an MIC value 500 μg/mL and an IZ of 10 μg/mL. According to the SAR study for all compounds, sydnone with 4-nitro benzothiazole had good efficacy against all bacterial and fungal strains [104].

## 11. Anti-Tubercular Activity

**Figure 8:**
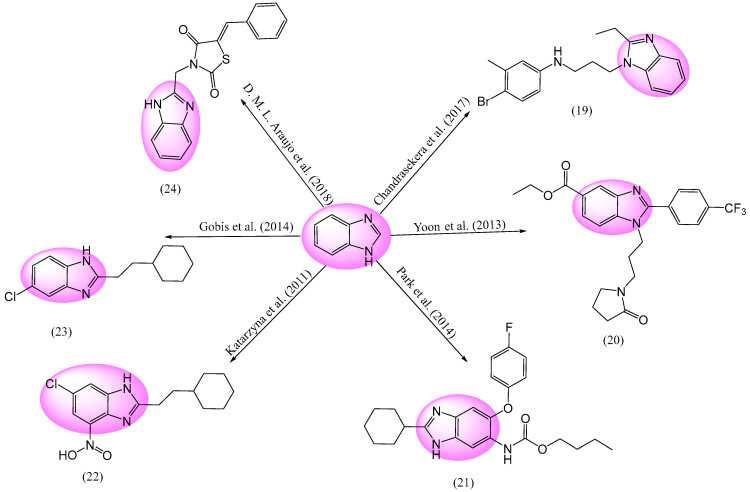
BnZ-based anti-tubercular activity [108,109,110,111,112,113,114,115,116,117].

The 2nd second-leading reason for mortality globally is tuberculosis (TB), a bacterial infection, and illness due to mycobacterium tuberculosis (M. tuberculosis) [105]. The present scenario to treat TB is DOTS (directly observed therapy short-course), which is the prescription of a mixture of three to four medicines for between six and twelve months [106,107]. These contain isoniazid, and ethambutol, etc. Figure 8 depicts a variety of anti-tubercular drugs with BnZ nuclei. A number of compounds of phenoxy alkyl-BnZ were discovered by Chandrasekera et al. A minimal inhibitory concentration (MIC)99 is needed to totally suppress *M. tuberculosis* formations in aqueous culture. Entity 19 (4-bromo-N-(3-(2-ethyl-1H-benzo[d]imidazol-1-yl)propyl)-3-methylaniline) was discovered to be the most effective compound, having an MIC of 52 μM [108]. Yoon et al. discovered a number of BnZ-based derivatives, including compounds examined in in vitro assay, that were modified from the microdilution of AlamarBlue (AB) complex 20 (ethyl-1-(3-(2-oxopyrrolidin-1-yl)propyl)-2-(4-(trifluoromethyl)phenyl)-1H-benzo[d]imidazole-5-carboxylate) (m.p. is 99–100 °C). It was discovered to be a highly effective anti-mycobacterial agent against *Mycobacterium tuberculosis* H37Rv, with an IC_50_ value of 11.52 μM. It was discovered that an electron-withdrawing group at the 4th position of the phenyl ring is significant for the activity of this compound [109].

Park et al. developed a method and synthesized a collection of 6, 5, or 2-tri-substituted BnZ variants, which later were evaluated for their effectiveness at treating TB. The efficacy of BnZ derivative 21 (butyl(2-cyclohexyl-5-(4-fluorophenoxy)-1H-benzo[d]imidazol-6-yl)carbamate) at preventing *Mycobacterium tuberculosis* filamentous temperature-sensitive mutant Z (Mtb-FtsZ) polymerization was tested [110]. The investigation of the chemically affected suppression of FtsZ polymerization was conducted using a light scattering test [111,112]. Entity 21 was discovered as a highly effective drug among all the produced compounds. Mtb Hinshaw 37, and Rockefeller university variant (H37Rv) strain were targeted by a variety of drugs, demonstrating effective MIC values in the range of 0.63–12.5 μg/mL [113]. Based on the procedure outlined by Katarzyna Gobis et al., complex 22 ((6-chloro-2-(2-cyclohexylethyl)-1H-benzo[d]imidazol-4-yl)(oxo)-l4-azanol) was produced via the heating of 3-cyclohexylpropanoic acid with 5-chloro-3-nitrobenzene-1,2-diamine, resulting in a yield of light yellow crystals. Compound 22 showed considerable anti-tuberculostatic action against *M. tuberculosis,* ranging between 1.5 and 12.5 mg/mL and having an m.p. of 190–192 °C [114]. In the study of Gobis et al, compound 23 (5-chloro-2-(2-cyclohexylethyl)-1H-benzo[d]imidazole) was produced by the heating of 3-cyclo-hexylpropanoic acid with the required di-amines. Again, component 23 was identified as the most effective antituberculosis molecule against *Mycobacterium tuberculosis* (M. TB) and *Mycobacterium bovis* (M. bovis), with an assessed MIC range from 0.75 to 1.5 mg/mL. Molecules having cyclohexyl ethyl substituents at the C-2 position are highly active compared to compounds with a longer chain [115]. *Mycobacterium tuberculosis* H37Rv strain was used to test the in vitro antitubercular activity of drug 24 ((Z)-3-((1H-benzo[d]imidazol-2-yl)methyl)-5-benzylidenethiazolidine-2,4-dione), which displayed a potent Mtb inhibitory effect, with an MIC of 6.25 g/mL. Compound 24 was believed to be the most potent analogue against mycobacterium, with equal efficiency against TB as the common antibacterial combination of streptomycin 2-(Chloromethyl)-1H-benzo[d]imidazole and thiazolidine-2,4-dione. This dual combination tends to give a greenish yellow color after 71 h. of reaction in acetonitrile, with a yield of 82% and an m.p. of 210 °C. TLC was used to observe the response of dry liquid, followed by the dilution of the residue with water, drying, and treatment with diethyl ether, to obtain the required components [116,117].

## 12. Anti-Protozoal Activity

**Figure 9:**
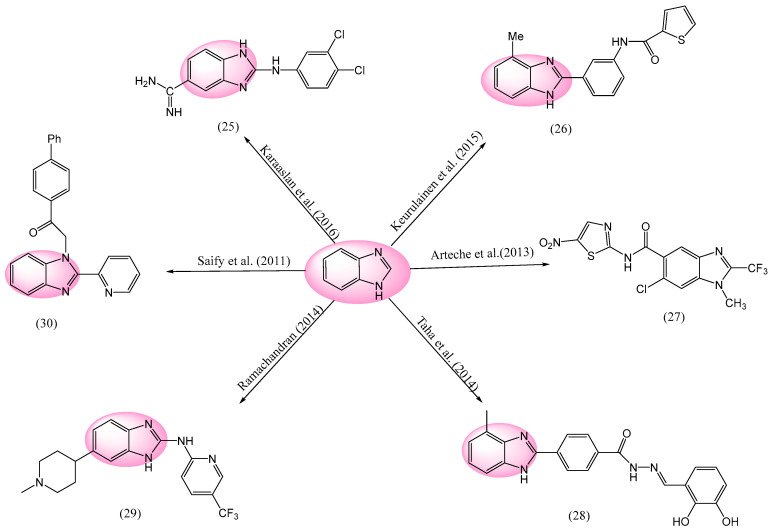
BnZ-based anti-protozoal activity [121,122,123,124,125,126].

Parasitic diseases are a significant threat to global health. Diseases that cause diarrhea, which are classified as one of the top five killers globally, include several gastrointestinal infections. Each of the global population suffers by one or more unnoticed tropical illnesses yearly, with around 1.6 million persons per day [118,119,120]. Figure 9 depicts some anti-protozoal medicines with BnZ-based moieties. The compound 2-anilinoBnZ was synthesized by Karaaslan et al, and the anti-protozoal potency of this drug was assessed. Compound 25 (2-((3,4-dichlorophenyl)amino)-1H-benzo[d]imidazole-5-carboximidamide) was examined in vitro for its anti-parasitic effect against *T. b. rhodesiense*, i.e., the bloodstream forms of trypomastigote and *P. falciparum* at erythrocytic stages [121]. Compound 25 displayed an IC_50_ value of 0.138 ± 0.017 μM against plasmodium falciparum, indicating a strong anti-malarial activity. In contrast, it also exhibited an IC_50_ value of 21.80 ± 8.01 μM against Trypanosoma brucei rhodesiense, indicating moderate activity against this parasite. Additionally, a cytotoxicity of compound 25, with an IC_50_ value of 39.06 ± 0.47 μM, was demonstrated against L6 cells (rat skeletal muscle cells), which indicates that this compound exhibited some toxicity toward these cells at higher concentrations. Drug 26 (N-(3-(4-methyl-1H-benzo[d]imidazol-2-yl)phenyl)thiophene-2-carboxamide), i.e., 2-arylBnZ, was synthesized by Keurulainen et al. It was effective against Tamm–Horsfall protein 1 (THP-1) cells infected with Leishmania donovani (L. donovani), displaying a 46% parasite reduction at 5 μM. The compounds 4- and 5-methylbenzimidazole variants displayed intriguing SARs. They showed comparable IC_50_ values and suppressed axenic amastigotes at all doses, but their effects on THP-1 cells were quite different. While 4-methyl derivative showed considerable action, the 5-methyl variant essentially demonstrated little effect [122].

Numerous BnZ-based compounds were discovered by Arteche et al., which were further tested for their antiprotozoal effects. Compound 27 (6-chloro-1-methyl-N-(5-nitrothiazol-2-yl)-2-(trifluoromethyl)-1H-benzo[d]imidazole-5-carboxamide) induced good antiprotozoal action against T. vaginalis, E. histolytica, and G. intestinalis, exhibiting IC_50_ values of 0.041 ± 0.005, 0.006 ± 0.002, and 0.021± 0.005 μM, respectively. This activity was increased by the addition of a methyl or a trifluoromethyl group at the 2 position of the BnZ nucleus [123]. The highly effective anti-leishmanial compound 28 ((E)-N′-(2,3-dihydroxybenzylidene)-4-(4-methyl-1H-benzo[d]imidazol-2-yl)benzohydrazide) was synthesized by Taha et al., which had an IC_50_ value of 37.80 ± 2.5 μM. The activity of the SAR is mostly influenced by the number and location of hydroxyl groups. The di-hydroxy substituents in drug 28 had outstanding activity, with variations in their action. The least active molecule among the di-hydroxy analogues is compound 28 (IC_50_ = 220.15 ± 1.95 μM) because it has high intra-molecular H-bonding, which reduces the activity of the active component methylglyoxal. Compound 28 was tested in vitro for its efficacy to scavenge DPPH radicals. It displayed a variable DPPH radical-scavenging activity of 12.05 ± 0.45, respectively [124]. Ramachandran et al. designed and evaluated a novel family of naryl-2-amino-benzoimidazol 29 named (6-(1-methylpiperidin-4-yl)-N-(5-(trifluoromethyl)pyridin-2-yl)-1H-benzo[d]imidazol-2-amine) for its anti-malarial activity against *P. falciparum*. Compound 29 was demonstrated to being highly effective, with an IC_50_ value of 36 μM. It was also easily available and efficient in a rat version of malaria. Furthermore, this series of drug exhibited a highly efficient death rate comparable to chloroquine in a unique in vitro study of its parasite-reduction ratio. More new clinical candidates from this chemical family should be ascertained, possibly by additional lead optimization attempts in combination with in vivo unsafe analysis [125]. The in vitro anti-plasmodial effects of a variety of BnZ derivatives, synthesized by Saify et al., were assessed against human Cytidine triphosphate D (CTP D) and *P. falciparum* plasmepsin II. Compound 30 (1-([1,1′-biphenyl]-4-yl)-2-(2-(pyridin-2-yl)-1H-benzo[d]imidazol-1-yl)ethan-1-one) demonstrated substantial anti-plasmodial action, with an IC_50_ value of 160 μM. Cathepsin D is inhibited by compound 30, with an inhibitory potency and selectivity comparable to that of plasmepsin II (IC_50_ = 2.0 μM vs. 14.7 μM). According to SAR studies, the additional phenyl group at the p-position of the acetophenone cause the moiety mosque’s enhanced anti-plasmodial action [126].

## 13. Conclusions

In conclusion, BnZ and its derivatives have been the focus of extensive investigations of their biological applications in several research areas, including their potential to treat cancer, reduce inflammation, fight against bacteria, treat tuberculosis, and inhibit protozoal growth. Green, non-metallic, and metallic methods have been used to synthesize BnZ and its derivatives. The desired product, as well as economic and environmental factors, all have an important role in the selection of the synthesis route. Recent research has placed a lot of emphasis on the creation of BnZ derivatives with enhanced biological activity, highlighting the potential of these substances as prospective medicines for treating different disorders. So, additional research is required to understand the mechanisms of action of these substances to enhance their biological activities.

## Data Availability

Not applicable.
